# Comparison of demographics and outcomes of patients with severe sepsis admitted to the ICU with or without septic shock

**DOI:** 10.1186/cc12948

**Published:** 2013-11-05

**Authors:** Bárbara Magalhães Menezes, Fernanda Vilas Bôas Araújo, Fábio Ferreira Amorim, Adriell Ramalho Santana, Felipe Bozi Soares, Jacqueline Lima de Souza, Mariana Pinheiro Barbosa de Araújo, Louise Cristhine de Carvalho Santos, Pedro Henrique Gomes Rocha, Mateus Gonçalves Gomes, Osvaldo Gonçalves da Silva Neto, Pedro Nery Ferreira Júnior, Alethea Patrícia Pontes Amorim, Rodrigo Santos Biondi, Rubens Antônio Bento Ribeiro

**Affiliations:** 1 Escola Superior de Ciências da Saúde, Brasília, Brazil; 2 Liga Acadêmica de Medicina Intensiva de Brasília, Brazil; 3 Hospital Anchieta, Brasília, Brazil

## Background

Severe sepsis and septic shock are common and are associated with substantial mortality and substantial consumption of healthcare resources [1]. Although the incidence of septic shock has steadily increased during the past several decades, the associated mortality rates have remained constant or have decreased only slightly [2]. This study aims to compare demographics and outcomes of patients admitted to the ICU with severe sepsis and with or without septic shock.

## Materials and methods

The present study is a retrospective cohort conducted over a 3-year period in the ICU of Hospital Anchieta, Brasília, Brazil. Patients were divided into two groups: severe sepsis without shock septic (SW) and severe sepsis with septic shock (SS). The patients coming from other ICUs or transferred to other ICUs were excluded.

## Results

A total of 198 patients with severe sepsis were enrolled in this study. Among them, 97 patients (49%) had septic shock. In this cohort, the mean age was 59 ± 16 years, the SAPS 3 score was 63 ± 17 and the APACHE II score was 21 ± 9. The mortality in four days was 12.6% (*n *= 25), in 28 days was 14.1% (*n *= 28) and the hospital mortality was 29.3% (*n *= 58). There was no difference between the two groups regarding age (64 ± 20 vs. 59 ± 21, *P *= 0.08) and length of stay in the ICU (12 ± 1 vs. 11 ± 1, *P *= 0.51). The SS group presented higher SAPS3 (70 ± 17 vs. 57 ± 15, *P *= 0.00) and APACHE II (1 vs. 8 ± 9 ± 1, *P *= 0.00) scores. Patients in the SS group also had higher mortality in 4 days (18% vs. 8%, *P *= 0.04), in 28 days (20% vs. 9%, *P *= 0.03) and hospital mortality (37% vs. 22%, *P *= 0.02).

## Conclusions

Patients admitted with septic shock had higher mortality than patients admitted with severe sepsis without septic shock, but there was no difference between the groups with respect to length of stay in the ICU.
